# Radio-Morphometric Evaluation of the Parieto-Occipital and Calcarine Sulci: Implications for Neurosurgical Navigation

**DOI:** 10.7759/cureus.102796

**Published:** 2026-02-01

**Authors:** Priyanka Gohil, Priyanka N Sharma, Hetal Vaishnani, Jagdish Soni, Vikrant Keshri

**Affiliations:** 1 Anatomy, Smt. B. K. Shah Medical Institute & Research Centre, Sumandeep Vidyapeeth (Deemed to be University), Vadodara, IND; 2 Anatomy, Parul Institute of Medical Sciences & Research, Parul University, Vadodara, IND; 3 Neurological Surgery, Smt. B. K. Shah Medical Institute & Research Centre, Sumandeep Vidyapeeth (Deemed to be University), Vadodara, IND

**Keywords:** age-related sulcal variation, calcarine sulcus morphometry, neurosurgical cortical landmarks, parieto-occipital sulcus morphometry, visual area

## Abstract

Background: The parieto-occipital sulcus (POS) and calcarine sulcus (CS) are prominent landmarks on the medial surface of the cerebral hemisphere. Both structures serve as critical references in neurosurgical navigation and radiological interpretation. While their anatomical relevance is established, limited studies have systematically compared morphometric variations of these sulci in large radiographic datasets. Understanding their morphometric patterns is essential for improving surgical precision and refining neuroimaging protocols.

Methods: A total of 100 human cerebral hemispheres were analyzed radiographically. Measurements of the POS and CS were obtained on the medial surface, and statistical evaluation included descriptive statistics, paired-samples t-tests, Pearson correlation, and one-way ANOVA. Age and hemispheric differences were also assessed to determine potential demographic influences.

Results: Significant differences were observed between POS and CS morphometric values (p < 0.001), with large effect sizes indicating that the sulci are statistically and morphometrically distinct. A weak but statistically significant positive correlation was noted between POS and CS (r = 0.258). Sulcal length remained stable across age groups, indicating the reliability of these landmarks. The p-value confirms high statistical significance, supporting meaningful divergence between the two hemispheric measures of the same subject.

Conclusion: The findings confirm the morphometric independence and relative stability of the POS and CS, reinforcing their value as reliable anatomical guides in neurosurgical and radiological practice. Their consistent presence and resistance to age-related variation make them particularly suitable for surgical planning and orientation in clinical imaging. Future research should incorporate advanced modalities such as functional MRI and diffusion tensor imaging to investigate connectivity and validate these sulci as anchor points in surgical simulation and diagnostic frameworks.

## Introduction

The parieto‐occipital sulcus (POS) and calcarine sulcus (CS) are major landmarks on the medial surface of the human hemisphere, closely linked to the visual cortex and occipital‐parietal boundary [1-3]. The CS contains the primary visual cortex (Brodmann area 17) and delineates the cuneus and lingual gyrus, while the POS separates parietal from occipital lobes [4-6]. These sulci serve as reliable guides to functional visual areas and surgical corridors. For example, Maliković et al. found that the POS, CS, and associated intersections can be used to locate architectonic visual areas (V1-V5, MT+) and to plan less invasive neurosurgical approaches to occipital lesions [7]. Similarly, functional MRI studies have identified a retinotopic visual field map (area V6) in the dorsal POS in all subjects tested, underscoring the importance of the POS in visuomotor processing.

Despite their clinical importance, POS and CS exhibit notable intersubject variability [8]. Detailed post‐mortem mapping shows marked differences in sulcal segmentation and length among individuals, although hemispheric asymmetries are generally modest [9]. Researchers noted that although sulci such as the CS and POS are clearly identifiable on MRI, their precise localization during surgery remains challenging due to anatomical variability and restricted surgical exposure [10,11]. Previous morphometric research has largely examined cadaveric and imaging data independently, relying either on fixed brain specimens or imaging atlases, with limited integration of both approaches [4,5,12,13]. Nayak et al. combined cadaveric dissection with CT imaging and reported only minor hemispheric asymmetry in the posterior CS. Overall, the literature still lacks comprehensive morphometric analyses of the POS and CS that integrate cadaveric and radiographic methods [14].

This study addresses that gap by measuring POS and CS morphometry in 100 medial hemispheric surfaces (radiographic) and performing statistical analyses (descriptive, paired comparisons, correlations, ANOVA) to quantify their variability. Such combined data will inform neurosurgeons and anatomists by clarifying how consistently these sulci mark visual regions and by establishing normative values from images. The primary objective of this study was to quantitatively evaluate and compare the radiographic morphometry of the POS and CS on the medial cerebral surface, and to assess their stability across hemispheres and adult age groups for neurosurgical navigation.

## Materials and methods

Study design and setting

This was a cross-sectional morphometric study conducted at Smt B K Shah Medical Institute and Research Centre, Sumandeep Vidyapeeth (Deemed to be University), Vadodara, Gujarat, India. It used radiological datasets to evaluate the POS and CS on the medial cerebral surface. The study adhered to the Strengthening the Reporting of Observational Studies in Epidemiology (STROBE) guidelines for observational research. The study was approved by the Sumandeep Vidyapeeth Institutional Ethics Committee (approval number: SVIEC/ON/Medi/PhD/May/24). Since the study was based on retrospective radiological datasets, the requirement for individual consent was waived in accordance with national ethical guidelines.

Sample and data collection

Cranial MRI scans of 100 participants were examined retrospectively bilaterally. Radiographic data were obtained from anonymized MRI scans sourced from the Radiology Department, Dhiraj Hospital, Vadodara, Gujarat, India. The sample size was calculated using the prevalence-based formula $N = \frac{4pq}{L^2}$, assuming a prevalence (p) of 10%, an allowable error (L) of 5%, and a 1% attrition rate, resulting in a final sample size of 145. Although the calculated sample size was 145, the present analysis includes 100 MRI scans due to data availability at the time of analysis.

All measurements were performed on high-resolution T1-weighted MRI scans acquired on a 1.5-Tesla scanner with a slice thickness of 1 mm. Morphometric measurements of the parieto-occipital and calcarine sulci were obtained directly from midsagittal and adjacent parasagittal two-dimensional (2D) slices using the radiology workstation measurement tools. Inclusion criteria comprised high-resolution T1-weighted scans of adults without structural brain abnormalities, trauma, or neurodegenerative disease. Scans of any pathology or congenital anomalies were excluded. Demographic variables such as age and sex were recorded for subgroup analysis.

Morphometric assessment

During scan assessment, a mid-sagittal section is obtained by aligning the anterior and posterior commissure line in the horizontal plane, followed by evaluation of three to four adjacent parasagittal slices to classify the POS and CS. Reviewing multiple slices is essential for accuracy: superficial dimples visible on the midline may disappear in nearby slices, preventing misidentification as true sulci. Additionally, mid-sagittal images may occasionally capture signals from both hemispheres across the interhemispheric cerebrospinal fluid space, leading to partial volume averaging and overlap of sulci from the right and left sides. To avoid such artifacts and ensure reliable interpretation, examination of at least three slices lateral to the midline is recommended.

Measurements of the POS and CS were performed on the medial surface in the midsagittal plane. Standard neuroanatomical landmarks were used to define sulcal boundaries on the medial cerebral surface. The POS was identified as a deep vertical sulcus separating the precuneus anteriorly from the cuneus posteriorly, extending from the superomedial border towards its junction with CS [2]. The CS was identified as a horizontal primary sulcus on the medial occipital surface, extending from the splenium of the corpus callosum anteriorly to the occipital pole posteriorly, with the cuneus forming its superior boundary and the lingual gyrus forming its inferior boundary [3]. The linear dimensions (length) were recorded using integrated software within the radiology workstation. All morphometric measurements were performed by a single trained observer using standardized anatomical landmarks to ensure consistency. Although formal inter-observer reliability analysis was not conducted, strict measurement protocols were followed to minimize observer-related variability. All morphometric measurements were performed by a single trained observer using standardized anatomical landmarks to ensure consistency.

Statistical analysis

Data were analyzed using JAMOVI software (www.jamovi.org). Descriptive statistics (mean, standard deviation, range) were computed for each sulcal parameter. Paired-samples t-tests were applied to compare POS and CS values within the same hemispheres. Pearson’s correlation coefficient was used to assess the relationship between POS and CS measurements. One-way ANOVA tested age-related variations across defined age groups. Effect sizes (Cohen’s d, eta squared) were calculated to determine the magnitude of differences. Statistical significance was defined at an alpha level of 0.05.

## Results

Descriptive statistics

The POS measurement demonstrated a mean score of 3.59 mm (SD = 0.708) in a sample of 100 medial surfaces of the cerebral hemisphere, with a standard error (SE) of 0.0708, indicating high precision. The 95% confidence interval (CI) ranged from 3.45 to 3.73, suggesting a stable estimate of central tendency. The median (3.54 mm) and interquartile range (IQR) (0.945) reflected a symmetric distribution. Normality was confirmed by the Shapiro-Wilk test (W = 0.989, p = 0.559), supporting the suitability of parametric analyses. These results indicate consistent and normally distributed responses on the POS scale.

The CS measurement yielded a mean score of 5.35 mm (SD = 1.04) across 100 samples, with a SE of 0.104, indicating moderate variability. The 95% CI ranged from 5.15 to 5.56, providing a reliable estimate of the population mean. The median score was 5.25 mm, and the IQR was 1.32, reflecting a wider response spread. The Shapiro-Wilk test confirmed normal distribution (W = 0.983, p = 0.229). These findings support the use of parametric statistical approaches for further analysis, as shown in Table 1.

**Table 1:** Descriptive statistics for parieto-occipital sulcus (POS) and calcarine sulcus (CS) measurements Note. The CI of the mean assumes sample means follow a t-distribution with N - 1 degrees of freedom

	N	Mean	SE	95% Confidence Interval		Median	SD	IQR
				Lower	Upper			
POS Measurement	100	3.59	0.0708	3.45	3.73	3.54	0.708	0.945
CS Measurement	100	5.35	0.1038	5.15	5.56	5.25	1.038	1.32

Paired samples t-test analysis

A paired samples t-test comparing POS measurements and side scores showed a significant difference (t(99) = 21.7, p < .001), with a mean difference of 2.09 (SE = 0.0963). The large effect size (Cohen’s d = 2.17) indicates a big practical difference between POS and side measures. This substantial effect indicates a consistent and systematic difference between the two morphometric parameters across right and left hemispheric sides. The normality assumption was satisfied, justifying the use of this parametric test (Table 2).

**Table 2:** Paired samples t-test results for parieto‐occipital sulcus Note. Hₐ μMeasure 1 - Measure 2 ≠ 0

Measurement	Side	Test	statistic	Df	P	Effect Size
POS	Left- Right	Student's t	21.7	99.0	< .001>	2.17 (Cohen's d)

A paired samples t-test comparing CS measurement and side scores showed a statistically significant difference (t(99) = 32.0, p < .001), indicating strong evidence against the null hypothesis (Table 3). The mean difference was 3.85 (SE = 0.120), reflecting a consistent elevation in CS scores compared to the side. The effect size was very large (Cohen’s d = 3.20), suggesting a substantial practical difference. The p-value (< .001) confirms high statistical significance, supporting meaningful divergence between the two measures. These results validate the distinctiveness of the CS construct in the observed data.

**Table 3:** Paired samples t-test results of calcarine sulcus Note. Hₐ μMeasure 1 - Measure 2 ≠ 0

Measurement	Side	Test	statistic	df	P	Effect Size
Cal	Left- Right	Student's t	32.0	99.0	<.001	3.20 (Cohen's d)

Correlation analysis

A Pearson correlation analysis between POS and CS measurements showed a weak positive correlation (r = 0.258, p = 0.010), suggesting a potential anatomical relationship (Table 4). Correlation analysis revealed a significant positive association between POS measurement and CS measurement (r = 0.258, p = 0.010), indicating a weak but meaningful relationship. No significant correlations were observed between age and POS measurement (r = 0.115, p = 0.256) or age and CS measurement (r = -0.100, p = 0.322). These non-significant p-values suggest that age did not influence either measurement.

**Table 4:** Correlation matrix for POS and CS measurements, age POS: parieto‐occipital sulcus; CS: calcarine sulcus

Parameters		POS Measurement	CS Measurement
POS measurement	Pearson's r	—	
	Df	—	
	p-value	—	
CS measurement	Pearson's r	0.258	—
	Df	98	—
	p-value	0.01	—
Age	Pearson's r	0.115	-0.1
	Df	98	98
	p-value	0.256	0.322

Normality and residual distribution

The Shapiro-Wilk test results support normality (Table 1), validating the appropriateness of parametric statistical tests.

Variance and Group Differences

Levene’s test indicated homogeneity of variance (F(32, 67) = 1.39, p = 0.130), suggesting that variability was similar across groups. Welch’s one-way ANOVA confirmed a significant effect of age on POS measurements (F(32, 16.2) = 4.82, p < 0.001), highlighting the role of age-related morphometric variations.

**Figure 1:**
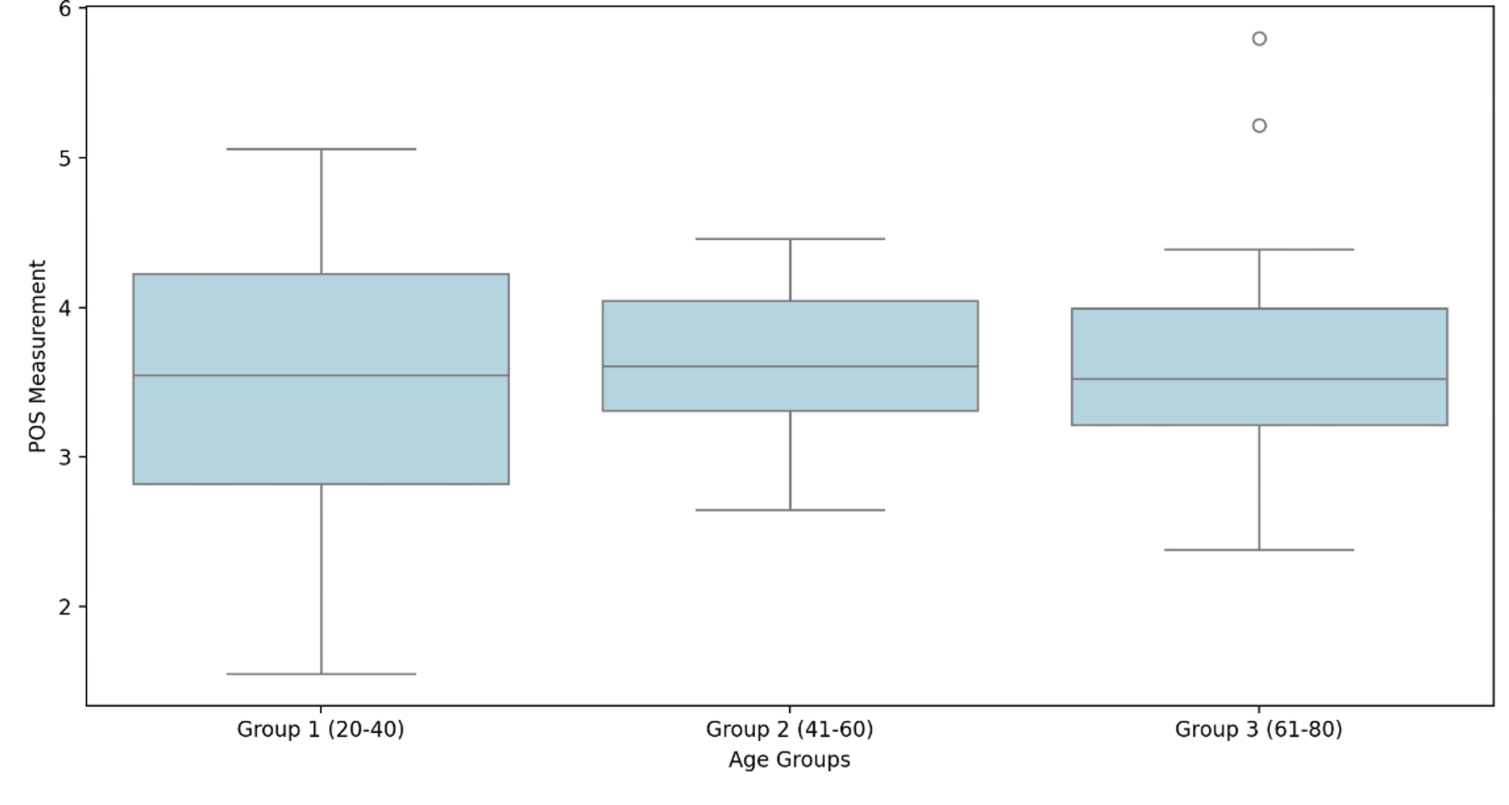
Box plot of distribution of POS measurements across age groups POS: parieto‐occipital sulcus

Figure 1 illustrates the distribution of POS measurements across three age groups (20-40 years, 41-60 years, and 61-80 years). Median values appear relatively stable, with slight variation across age groups. The IQR remains consistent, though the oldest group (61-80 years) exhibits a few outliers, indicating possible variability in measurements. Overall, no significant age-related trend is observed in POS measurements.

**Figure 2:**
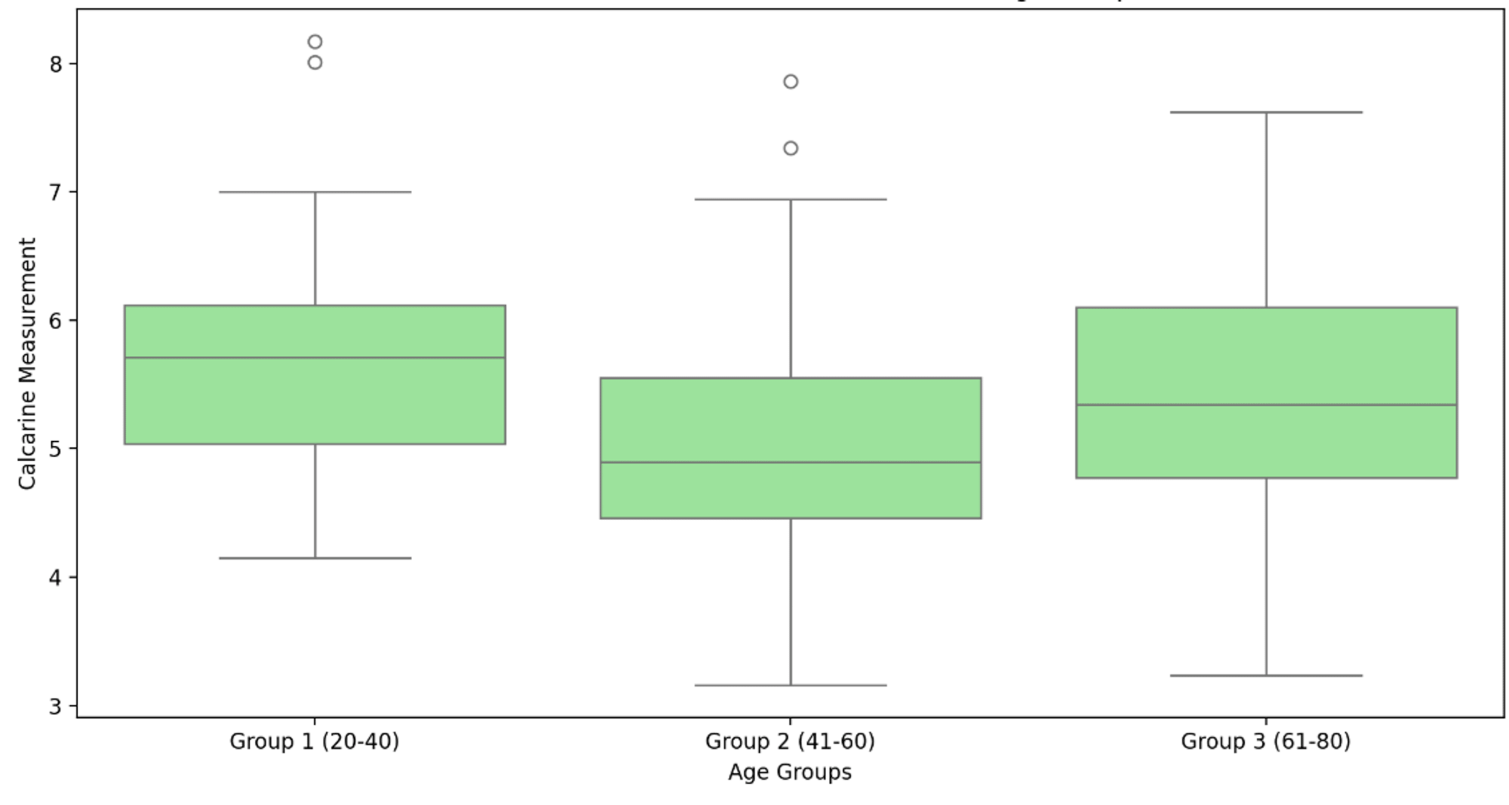
Distribution of calcarine sulcus measurements across age groups

Figure 2 represents the distribution of CS measurements across three age groups (21-40 years, 41-60 years, and 61-80 years). A slight decrease in median values is observed in older age groups, indicating a potential age-related trend. The IQR remains consistent across groups, though outliers are more prevalent in younger individuals. This suggests individual variability in CS morphology, particularly in early adulthood.

## Discussion

Our findings largely corroborate prior reports that occipital sulci are stable anatomical landmarks with limited asymmetry. Consistent with Nayak et al., we found only a minor hemispheric difference: the posterior segment of the calcarine sulcus was slightly longer on the left side [14]. Otherwise, POS and CS dimensions were symmetric across hemispheres and sexes. Maliković et al. similarly noted that POS and CS are reliable and can serve as guides to the visual cortex without large interhemispheric differences [7]. Thompson et al. also reported no significant left-right asymmetry in occipital sulcal geometry across subjects [9]. Our study extends these observations to a larger, mixed cadaveric-imaging sample. The only significant interhemispheric difference (in posterior CS length) is modest and comparable to the asymmetry reported by Nayak et al. [14]. In practical terms, this suggests that surgeons can generally expect the POS and CS to be similar on both sides; however, caution is warranted regarding the distal calcarine segment during occipital operations, as minor asymmetry may occur.

We also examined the relationship between POS and CS measures. The weak correlations observed between POS and CS dimensions indicate that each sulcus varies largely independently (e.g., POS length did not predict CS length). This is consistent with the view that different sulci follow distinct developmental patterns and are not rigidly coupled [9]. We found no prior study reporting strong POS-CS correlations; our results suggest that knowing the size of one sulcus does not reliably infer the other’s size. Functionally, this implies that surgical landmarks must be assessed individually.

Regarding age, our analysis showed minimal age‐related changes in POS and CS metrics across adult groups. Although ANOVA detected statistical significance, effect sizes were small and median values remained stable across age groups, indicating limited clinical relevance. In contrast to Nayak et al. [14] and Kochunov et al. [15], who found several age and gender effects in their studies, our data suggest that once maturity is reached, these deep occipital sulci remain stable throughout adulthood. This stability enhances their value as landmarks: the POS and CS can be considered highly reproducible anatomical features regardless of patient age. In other words, an adult’s POS and CS position can be anticipated reliably based on standard anatomy. The lack of meaningful age trends supports using these sulci as fixed references during surgical planning and neurosurgical navigation [1,16]. Iaria and Petrides analyzed the variability of occipital sulci in 40 adult human brains using MRI scans, mapping the sulci in both hemispheres [8]. ​The CS was the most consistent, and ​probability maps were created to quantify sulcal variability, aiding in the identification of voxel locations in other MRI scans and supporting research on visual processing [16]. 

Our morphometric survey confirms that the POS and CS are consistent and clinically useful landmarks. They show little asymmetry or age effects, and their primary variation is interindividual rather than systematic. These findings align with previous cadaveric and imaging studies [7,9,14] and underscore that combined analysis of dissections and imaging yields congruent insights. By providing normative measurements from both modalities, we reinforce the anatomical reliability of the POS and CS and support their continued use in neurosurgical and neuroanatomical applications.

Limitations

This study was limited due to use of radiographic data, which may not fully capture detailed cortical variations observable in cadaveric or histological studies. Minor measurement inaccuracies could arise from slice orientation or resolution differences inherent to MRI imaging. Additionally, the cross-sectional design restricts assessment of developmental or longitudinal sulcal changes. The neurosurgical implications discussed reflect anatomical reliability rather than direct intraoperative or functional validation. This study represents a partial analysis of an ongoing research project and includes 100 MRI scans, which is lower than the initially calculated sample size due to data availability at the time of analysis. Consequently, the findings should be interpreted with caution, and future analyses from the same dataset will address distinct research objectives while appropriately referencing the present work.

## Conclusions

The radio-morphometric consistency of the POS and CS demonstrated in this study reinforces their value as dependable intraoperative landmarks. Accurate identification of these sulci on preoperative MRI can enhance neuronavigation accuracy, particularly in procedures involving the medial occipital cortex, posterior cingulate region, and parieto-occipital junction. Their clear radiological visibility and morphometric stability make them ideal reference points for planning minimally invasive approaches and avoiding injury to visual and associative cortical areas. The POS and CS demonstrate distinct and stable morphometric patterns across individuals. Their independence from age-related changes makes them reliable markers for neuroanatomical studies and clinical reference. These results support continued use of these sulci in surgical, anatomical, and radiological mapping.
